# Smoking-Related Interstitial Fibrosis (SRIF) in Patients Presenting With Diffuse Parenchymal Lung Disease

**DOI:** 10.1093/ajcp/aqac144

**Published:** 2022-12-10

**Authors:** Susan J Vehar, Ruchi Yadav, Sanjay Mukhopadhyay, Avantika Nathani, Leslie B Tolle

**Affiliations:** Pulmonary Critical Care and Sleep Medicine, University of Miami Miller School of Medicine, Miami, FL, USA; Imaging Institute, Cleveland Clinic, Cleveland, OH, USA; Department of Pathology, Pathology and Laboratory Medicine Institute, Cleveland Clinic, Cleveland, OH, USA; Respiratory Institute, Cleveland Clinic, Cleveland, OH, USA; Respiratory Institute, Cleveland Clinic, Cleveland, OH, USA

**Keywords:** Smoking-related interstitial fibrosis, Diffuse parenchymal lung disease, Interstitial lung disease, Respiratory bronchiolitis, Desquamative interstitial pneumonia

## Abstract

**Objectives:**

To describe the clinical, radiologic, and pathologic findings in cases where smoking-related interstitial fibrosis (SRIF) was diagnosed in surgical lung biopsy specimens from patients with clinical and imaging features of diffuse parenchymal lung disease (DPLD).

**Methods:**

Cases were included in this study if patients had clinical and imaging evidence of DPLD and surgical lung biopsy specimens revealed SRIF. A dedicated multidisciplinary conference was held to correlate clinical, radiologic, and pathologic findings.

**Results:**

Six cases met inclusion criteria; all six (five women/one man, aged 42-57 years, mean age 47 years) were either current smokers (five of six) or ex-smokers (one of six) and were evaluated for respiratory symptoms and abnormal pulmonary function tests, most commonly reduced forced vital capacity (n = 3) and diffusing capacity for carbon monoxide (n = 6). The most common imaging abnormalities were bilateral ground-glass opacities, which correlated with histopathologic SRIF. Follow-up of up to 10 years showed stable or improved clinical symptoms, pulmonary function tests, and radiologic findings with smoking cessation (three patients) or a decrease in smoking (three patients). No specific treatments were given, and those treated with empiric corticosteroid tapers did not show discernible responses.

**Conclusions:**

SRIF can present as clinically meaningful diffuse parenchymal lung disease in relatively young heavy smokers, characterized by bilateral ground-glass opacities and a stable clinical course.

**KEY POINTS**

- Although smoking-related interstitial fibrosis (SRIF) is a common incidental finding in lobectomies for cancer, it occasionally has clinicoradiologic features of interstitial lung disease.
- Patients with SRIF who have clinical evidence of interstitial lung disease are most commonly relatively young heavy smokers with abnormalities in forced vital capacity and diffusing capacity for carbon monoxide.
- Treatment of SRIF with corticosteroids does not appear to be effective. Long-term follow-up indicates stable or improved clinical symptoms and occasional pulmonary function test improvements with smoking cessation.

## INTRODUCTION

Smoking-related interstitial fibrosis (SRIF) is a pathologically defined form of interstitial fibrosis. The term SRIF was first introduced in 2010 by Katzenstein et al^1^ after careful review of extensively sampled lobectomy specimens in current and former smokers. The histologic hallmark of SRIF is expansion of alveolar septa by a distinctive form of eosinophilic, paucicellular fibrosis composed of “ropey-appearing” collagen.^2^ Since its initial description, SRIF has been recognized on the basis of its distinctive pathologic features in surgical lung resections from smokers.^3-7^ In addition, some reports have described identical histologic findings using alternative terminology such as “respiratory bronchiolitis-associated interstitial lung disease (RBILD) with fibrosis,”^8^ “respiratory bronchiolitis with fibrosis (RBF),”^9^ and “airspace enlargement with fibrosis.”^10^

Because the initial report described SRIF as a “clinically occult” finding, it has been generally assumed that SRIF is clinically insignificant. Based on this assumption, clinical and radiologic investigation of these patients has been limited. In 2016, Otani et al^7^ described the radiologic correlates of SRIF in 78 lobectomies, including low-attenuation areas, clustered cysts with visible walls, and ground-glass attenuation with or without reticulation. To our knowledge, the clinical manifestations and natural history of patients with SRIF have not been described in detail.

Anecdotally, we have encountered cases presented at our weekly multidisciplinary diffuse parenchymal lung disease (DPLD) conference in which SRIF was noted in surgical lung biopsies performed in patients being evaluated for DPLD. These patients had significant clinical symptoms and radiologic abnormalities, warranting not only referral to our center but also transbronchial and/or surgical lung biopsies. The aim of this study was to retrospectively review and describe the clinical, radiologic, and pathologic findings in these patients.

## MATERIALS AND METHODS

### Case Selection

This was a single-center retrospective study of patients referred to the Cleveland Clinic DPLD clinic for evaluation between 2013 and 2022. Our DPLD program holds a weekly multidisciplinary meeting with participation from physicians specialized in pulmonology, thoracic radiology, and pathology. Cases discussed are recorded in our DPLD database.

We included cases in this study if they fulfilled the following criteria: (1) the case was discussed at our multidisciplinary DPLD conference because of clinical and/or imaging features that raised concern for DPLD, (2) SRIF was diagnosed on surgical lung biopsy, and (3) both pathology slides and computed tomography (CT) scans were available for a focused multidisciplinary rereview.

In each case, all available imaging studies were rereviewed by a thoracic radiologist (R.Y.), and all available pathology specimens were rereviewed by a pulmonary pathologist (S.M.). Clinical, radiologic, and pathologic findings were then reviewed together in a focused, multidisciplinary discussion including a pulmonologist (S.J.V.), thoracic radiologist (R.Y.), and pulmonary pathologist (S.M.) to determine whether any consistent and clinically relevant patterns could be gleaned. Our review and discussion were performed in a nonblinded fashion.

This study was approved by the Institutional Review Board (IRB #21-709).

### Clinical Data

Electronic medical records were reviewed retrospectively for age, sex, smoking history, history of present illness, medical history, exposure/social history, type of lung biopsy specimen, pulmonary function testing, treatment, and follow-up.

### Radiographic Data

Available CT scans were reviewed by an expert thoracic radiologist (R.Y.). We used a semiquantitative scoring system to characterize the extent of involvement of lung parenchyma by key abnormalities, including emphysema, ground-glass opacities (GGOs), fibrosis, honeycombing, and cystic changes on visual assessment. The score ranged from 0 to 5 as follows: 0, abnormality is absent or minimal (involves <1% of lung parenchyma); 1, abnormality involves 1% to 5% of lung parenchyma; 2, abnormality involves 6% to 25% of lung parenchyma; 3, abnormality involves 26% to 50% of lung parenchyma; 4, abnormality involves 51% to 75% of lung parenchyma; and 5, abnormality involves more than 75% of lung parenchyma.

Additional pertinent radiographic features, including lymphadenopathy and bronchial wall thickening, were also noted.

### Histologic Data

Surgical lung biopsy specimens were reviewed by an expert pulmonary pathologist (S.M.). The same semiquantitative scoring system used by the radiologist was used by the study pathologist to characterize the extent of lung involvement by emphysema, pigmented airspace macrophages (respiratory bronchiolitis), and interstitial fibrosis.

## RESULTS

Six cases met the inclusion criteria and had clinically relevant patterns consistent with SRIF as a leading consideration.

### Clinical History and Pulmonary Function

All six patients were current (n = 5) or former (n = 1) smokers with a median of 27.5 pack-years of smoking (mean, 31.7 pack-years; range, 15-55 pack-years) at the time of biopsy. The baseline characteristics are shown in **TABLE 1**. Our patients were predominantly female (female/male = 5:1) with a median age of 45.5 years (mean, 47 years; range, 42-57 years) at the time of biopsy diagnosis. All six patients were evaluated for respiratory symptoms with modified Medical Research Council grade ranging from 1 to 4.

**TABLE 1:** Baseline Characteristics^a^

Feature	Cases (n = 6)
Age, y	
Mean	47
Median	45.5
Range	42-57
Sex	
Female	5 (83)
Male	1 (17)
Female/male ratio	5:1
Race	
White	5 (83)
Not reported	1 (17)
Smoking history	
Current/ever	6 (100)
Pack-years (median) Range	27.5 15, 55
Never	0 (0)
Body mass index	
Underweight (<18.5 kg/m^2^)	0 (0)
Normal weight (18.5-24.9 kg/m^2^)	1 (17)
Overweight (25-29.9 kg/m^2^)	2 (33)
Obese (>30.0 kg/m^2^)	3 (50)
Initial mMRC dyspnea score	
0	0 (0)
1	2 (33)
2	1 (17)
3	1 (17)
4	2 (33)
Comorbid conditions	
Asthma	1 (17)
COPD	
By ATS criteria	1 (17)
By GOLD criteria	2 (33)
CAD	1 (17)
DVT/PE	2 (33)
Cancer	0 (0)
Diagnostic procedure	
Bronchoscopy with biopsy	3 (50)
Three-lobe surgical lung biopsy	5 (83)
One-lobe surgical lung biopsy	1 (17)

ATS, American Thoracic Society; CAD, coronary artery disease; COPD, chronic obstructive pulmonary disease; DVT, deep vein thrombosis; GOLD, Global Initiative for Chronic Obstructive Lung Disease; mMRC, modified Medical Research Council; PE, pulmonary embolism.

^a^Values are presented as No. (%) unless otherwise indicated.

Spirometry findings are shown in **TABLE 2**. Using the Global Initiative for Chronic Obstructive Lung Disease criteria, two patients had obstruction on pulmonary function testing defined by a forced expiratory volume in the first second (FEV1)/forced vital capacity (FVC) ratio less than 0.7. Using American Thoracic Society criteria, one patient had baseline obstruction on pulmonary function testing as defined by a FEV1/FVC ratio less than the lower limit of normal with a corresponding FEV1%_predicted_ of 70%. All patients had an FEV1%_predicted_ of 70% or more with a median value of 72% and range of 70% to 92%. Three patients had an FVC below the lower limit of normal. For all patients, the median FVC%_predicted_ was 75%, with a range of 63% to 95%. Two of five patients (cases 3 and 4) demonstrated restriction based on total lung capacity; one patient did not have total lung volume recorded. Testing for diffusing capacity for carbon monoxide (DL_CO_) was performed in all six patients, revealing a median DL_CO_%_predicted_ of 53%, with a range of 33% to 68%.

**TABLE 2:** Pulmonary Function Testing at the Time of Initial Diagnosis in Patients With SRIF

PFT	Case 1	Case 2	Case 3	Case 4	Case 5	Case 6
FEV1, L						
Actual	3.2	2.2	2.2	2.2	1.8	2.3
LLN	3.2	2.6	2.4	2.5	2.0	1.9
% Pred	81	70	71	72	71	92
FVC, L						
Actual	4.7	3.2	2.4	2.7	2.2	3.0
LLN	4.1	3.2	3.1	3.1	2.6	2.5
% Pred	94	80	63	70	70	95
FEV1/FVC						
Actual	68	69	92	81	82	77
LLN	68	72	71	72	71	69
TLC, L						
Actual	6.4	5.2	3.2	3.9	Not available	4.3
LLN	5.1	4.4	4.3	4.1	Not available	3.6
% Pred	95	94	59	74	Not available	91
DL_CO_, mL/mm Hg, min						
Actual	12.3	16.7	8.03	14.4	12.4	10.4
LLN	23.2	18.0	17.3	17.5	15.2	14.6
% Pred	39	68	33	59	57	49
**Interpretation**	No obstruction or restriction; severely reduced DL_CO_	Moderate obstruction, no restriction; mildly reduced DL_CO_	No obstruction; restriction with severely reduced DL_CO_	No obstruction; restriction with moderately reduced DL_CO_	No obstruction; moderately reduced DL_CO_	No obstruction or restriction; moderately reduced DL_CO_

DL_CO_, diffusion capacity of carbon monoxide; FEV1, forced exhaled volume in 1 second; FVC, forced vital capacity; LLN, lower limit of normal; PFT, pulmonary function test; Pred, predicted; TLC, total lung capacity.

A summary of the clinical, radiologic, and pathologic findings along with treatment and follow-up data is shown in **TABLE 3**. In case 1, the possibility of combined pulmonary fibrosis and emphysema was raised by the pulmonologist prior to surgical lung biopsy based on “relatively normal spirometry with isolated low DL_CO_.” In case 6, the possibility of idiopathic pulmonary fibrosis (IPF) was raised after the surgical lung biopsy specimen was misdiagnosed as usual interstitial pneumonia (UIP) at another institution. The patient was subsequently seen at our institution for a second opinion, where a second review of the biopsy specimen revealed the correct diagnosis. This case highlights the fact that SRIF can be misdiagnosed as UIP because subpleural fibrosis can occur in both entities.

**TABLE 3:** Clinical, Radiologic, and Pathologic Features and Treatment/Follow-up in SRIF Presenting as DPLD^a^

Case No. Age, y/Sex	Clinical Features	Smoking	Imaging	Pathology	Treatment and Follow-up
1 46/M	Progressive dyspnea on exertion × 2 years; clubbing Differential diagnosis: sarcoidosis, RBILD, DIP, NSIP, CPFE, IPF	Ex-smoker—quit 3 months prior 45 PY (1.5 PPD × 30 years)	Bilateral symmetric, peripheral GGOs, diffuse but worse in the lower lobes, with bronchial wall thickening Trivial centrilobular and paraseptal emphysema present Mild mediastinal and hilar adenopathy (largest 2.2 cm, subcarinal)	Three-lobe surgical lung biopsy: SRIF with pigmented airspace macrophages; mild emphysema present Interstitial fibrosis and macrophages are diffuse (NSIP-like) in upper lobe >> middle and lower (very minimal, focal fibrosis, far fewer macrophages) Prior TBBX: RB and mild SRIF	Treatment: smoking cessation; patient did quit smoking; prednisone taper 30 mg × 1 month, 20 mg × 1 month, 10 mg × 1 month, stopped; supplemental oxygen Clinical follow-up (8 months): dyspnea on exertion persists Imaging follow-up (3 months): stable findings
2 44/F	Progressive dyspnea × 1 year History of factor V Leiden deficiency (discovered after MI at age 43) Differential diagnosis: chronic thromboembolic pulmonary hypertension	Current smoker 15 PY (0.5 PPD × 30 years)	Bilateral multifocal patchy GGOs noted predominantly in upper lobes, with bronchial wall thickening and mosaic attenuation No emphysema or thoracic adenopathy	Three-lobe surgical lung biopsy: SRIF with pigmented airspace macrophages; mild emphysema present Interstitial fibrosis and macrophages most prominent in upper lobe, much less (and very focal) in other lobes No prior TBBX	Treatment: Smoking cessation; patient did quit smoking; ICS-LABA Clinical follow-up (30 months): persistent exertional dyspnea, on home oxygen Imaging follow-up (9 months): stable findings
3 48/F	Cough, dyspnea, and wheezing × 5 months History of asthma (no treatment) Differential diagnosis: asthma, interstitial lung disease	Current smoker 32 PY (1 PPD × 32 years)	Bilateral diffuse GGOs with bronchial wall thickening, sparing the lung apices No emphysema Mild mediastinal and hilar adenopathy	Three-lobe surgical lung biopsy: SRIF with pigmented airspace macrophages; very mild emphysema present Fibrosis minimal, patchy, subpleural; macrophages variable but overall numerous Changes similar in all three lobes Prior TBBX: RB and mild SRIF	Treatment: Smoking cessation; patient continued to smoke; ICS-LABA Clinical follow-up (21 months): intermittent cough, wheezing Imaging follow-up (89 months): stable findings
4 42/F	Dyspnea × 1 year, productive cough, wheeze History of lupus; birds in home; worked as a welder Differential diagnosis: hypersensitivity pneumonitis, LIP	Current smoker 20 PY (1 PPD × 20 years)	Bilateral patchy lower lobe-predominant GGOs with bronchial wall thickening and mosaic attenuation Mild paraseptal emphysema present Mild mediastinal and hilar adenopathy	Three-lobe surgical lung biopsy: SRIF with pigmented airspace macrophages; mild emphysema present Fibrosis middle lobes > upper and lower Prior TBBX not available for review	Treatment: Prednisone 20 mg for 6 months with plan for taper; smoking cessation; patient did quit smoking Clinical follow-up (45 months): persistent dyspnea on walking and talking, on oxygen (3 L per minute during exertion) Imaging follow-up (48 months): stable findings
5 45/F	Recurrent pneumonias (three episodes), daily cough with expectoration, MRC grade 2 dyspnea × 1 year History of bipolar disorder, chronic low back pain on multiple psychotropic medications Differential diagnosis: bronchiectasis, ILD related to hypogammaglobulinemia, ILD related to smoking, sarcoidosis	Current smoker 23 PY (1 PPD × 23 years)	Bilateral multifocal, predominantly bronchocentric nodular GGOs with sparing of the lung bases (upper lobe predominant on right side) No emphysema Mild mediastinal and hilar adenopathy Radiologic impression: organizing pneumonia pattern	Three-lobe surgical lung biopsy: SRIF with pigmented airspace macrophages; fibrosis mild; macrophages numerous, diffuse; changes similar in all three lobes; mild emphysema present No prior TBBX. Mediastinoscopy negative for granulomas	Treatment: Smoking cessation advised (briefly stopped smoking around time of lung biopsy; later cut down from 1 PPD to ½ PPD but unable to quit); short courses of antibiotics and steroids for recurrent pneumonia, but longer corticosteroid course not given due to history of bipolar disorder and gastritis Clinical follow-up (118 months): persistent MRC grade 2 dyspnea, minimal cough; continues to smoke (1 PPD) Imaging follow-up (95 months): follow-up CT 4 years after initial CT showed complete resolution of nodular GGOs; stable changes almost 8 years later
6 57/F	Dyspnea × 14 months, cough × 8 months Differential diagnosis: idiopathic pulmonary fibrosis	Current smoker 55 PY (1.5 PPD × 30 years, then 1 PPD × 10 years)	Diffuse GGOs in the lower lobes with bronchial wall thickening and mosaic attenuation No emphysema Mild mediastinal and hilar adenopathy Radiologic impression: ?MAI	Single lobe (right lower lobe) surgical lung biopsy: SRIF with pigmented airspace macrophages; mild emphysema present No prior TBBX available	Treatment: Smoking cessation; patient cut back from 2 PPD to ½ PPD but did not quit completely Clinical follow-up (64 months): persistent MRC grade 1-2 dyspnea, cough; continues to smoke 1/2 PPD Imaging follow-up (46 months): stable findings

CPFE, combined pulmonary fibrosis and emphysema; CT, computed tomography; DIP, desquamative interstitial pneumonia; GGO, ground-glass opacity; ICS-LABA, inhaled corticosteroid–long-acting β-agonist; ILD, interstitial lung disease; IPF, idiopathic pulmonary fibrosis; LIP, lymphoid interstitial pneumonia; MAI, *Mycobacterium avium-intracellulare*; MI, myocardial infarction; MRC, Medical Research Council; NSIP, nonspecific interstitial pneumonia; PPD, packs per day; PY, pack-years; RB, respiratory bronchiolitis; RBILD, respiratory bronchiolitis-associated interstitial lung disease; SRIF, smoking-related interstitial fibrosis; TBBX, transbronchial lung biopsy.

^a^All ages and pack years are at time of biopsy-confirmed diagnosis of SRIF.

### Radiologic Data

**FIGURE 1:**
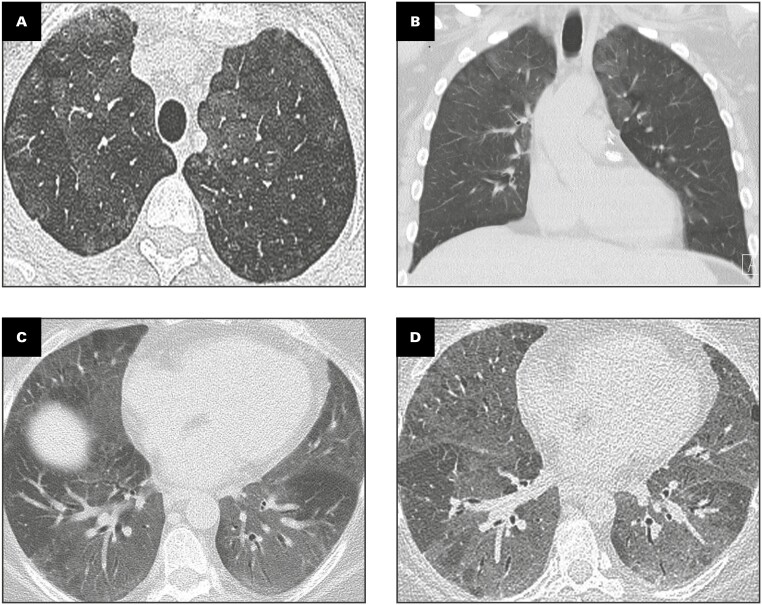
Radiologic findings in smoking-related interstitial fibrosis. **A**, **B**, Case 2 (44-year-old woman, 15 pack-year current smoker). High-resolution axial computed tomography (CT) (**A**) and nonenhanced coronal CT (**B**) demonstrate bilateral multifocal patchy ground-glass opacities in the upper lobes with bronchial wall thickening. **C**, **D**, Case 3 (48-year-old woman, 32 pack-year current smoker with forced vital capacity 63% predicted, diffusing capacity for carbon monoxide 33% predicted). Nonenhanced axial CT at diagnosis (**C**) and 89 months later (**D**) demonstrates diffuse ground-glass opacities in both lungs with bronchial wall thickening. Note stability of imaging abnormalities over more than 7 years.

**FIGURE 2:**
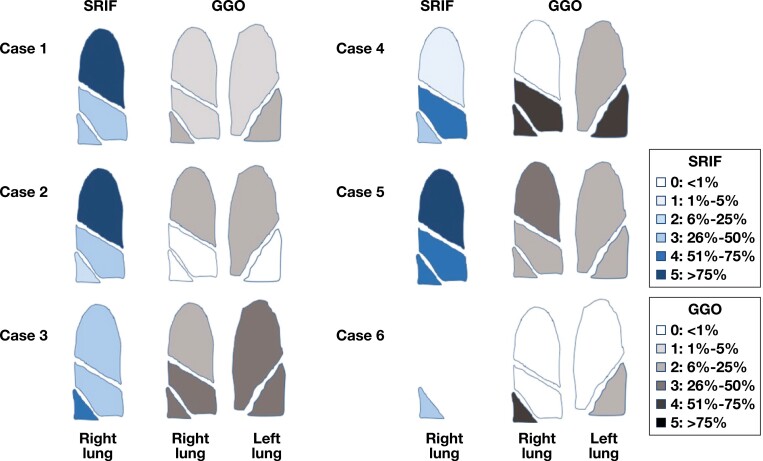
Correlation of ground-glass opacities (GGOs) and histopathologic smoking-related interstitial fibrosis (SRIF). Representation of right surgical lung biopsy SRIF and bilateral lung radiologic GGOs using a semiquantitative scoring system to describe tissue involvement. Of note, case 6 was a single lower lobe sample.

Radiologic features in SRIF are shown in **FIGURE 1**. Bilateral GGOs were the dominant feature in all cases and correlated with the presence of SRIF and pigmented airspace macrophages on histopathology, as shown in **FIGURE 2**. GGOs were usually present in a diffuse distribution, although they were upper lobe predominant in two cases (cases 2 and 5) and lower lobe predominant in two (cases 4 and 6). One case featured bronchocentric nodular GGOs mimicking organizing pneumonia (case 5). When present, GGOs were noted to affect 6% to 75% of the affected lobe. Mediastinal and/or hilar lymphadenopathy was noted in five cases.

**FIGURE 3:**
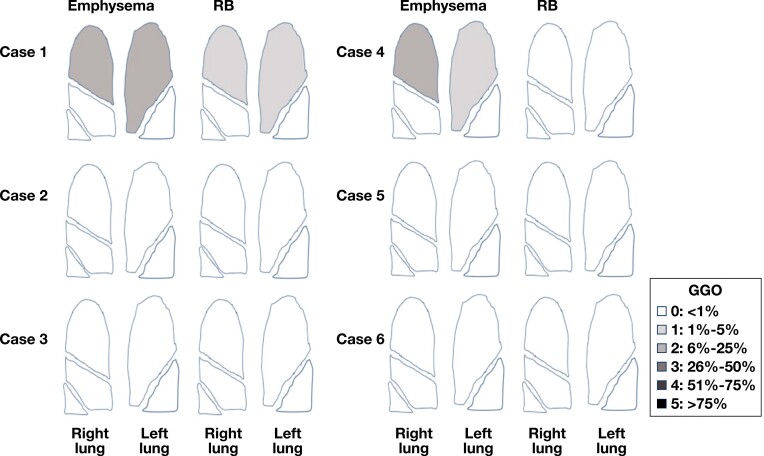
Radiologic semiquantification of emphysema and respiratory bronchiolitis (RB) in patients with smoking-related interstitial fibrosis. Representation of bilateral computed tomography findings of emphysema and RB using a semiquantitative scoring system. GGO, ground-glass opacity.

Radiologic semiquantification of emphysema and respiratory bronchiolitis for each case is shown in **FIGURE 3**. Emphysema was noted radiologically in only two of six cases (cases 1 and 4), where it involved less than 25% of bilateral upper lobes. Respiratory bronchiolitis was noted radiologically in only one case, involving less than 5% of both upper lobes. Radiologic evidence of fibrosis and honeycombing was absent in all cases.

### Histologic Data

**FIGURE 4:**
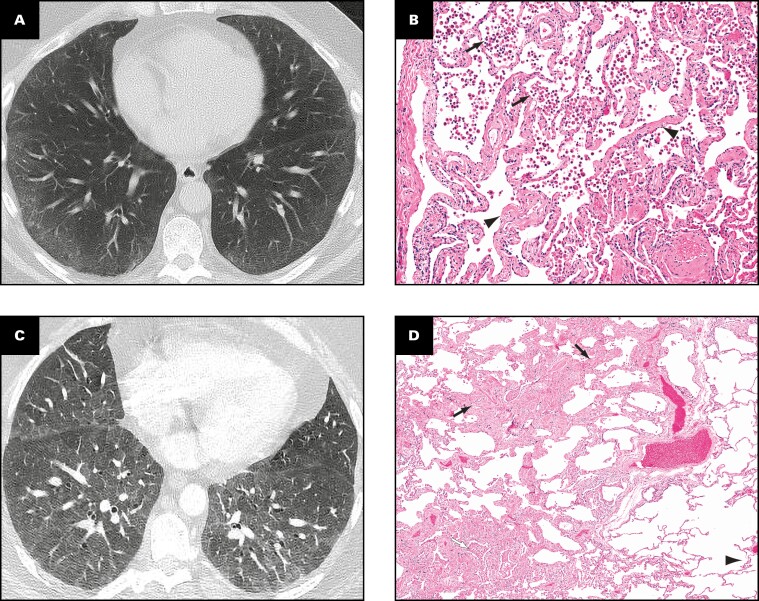
**A-D**, Smoking-related interstitial fibrosis: radiologic findings with pathologic correlates. **A**, A 42-year-old woman, 20 pack-year current smoker (case 4). Nonenhanced axial computed tomography (CT) demonstrates bilateral symmetric, peripheral, lower lobe–predominant ground-glass opacities with bronchial wall thickening. **B**, Surgical lung biopsy specimen, right lower lobe, from same patient. Alveolar septa are mildly thickened by “ropey” interstitial fibrosis (arrowheads). Arrows indicate filling of airspaces by numerous pigmented macrophages (H&E, ×10). **C**, A 46-year-old man, 45 pack-year ex-smoker (case 1). Enhanced axial CT demonstrates bilateral mid-to-lower lung-predominant patchy ground-glass opacities with bronchial wall thickening and mosaic attenuation. **D**, Pathology corresponding to **C**. Surgical lung biopsy specimen, right lower lobe. Alveolar septal thickening (interstitial fibrosis, black arrows) in this lobe is patchy. Note near-normal lung (arrowhead) adjacent to fibrotic areas. The airspaces in the fibrotic areas contain several macrophages (white arrow) but the nonfibrotic lung does not (H&E, ×4).

**FIGURE 5:**
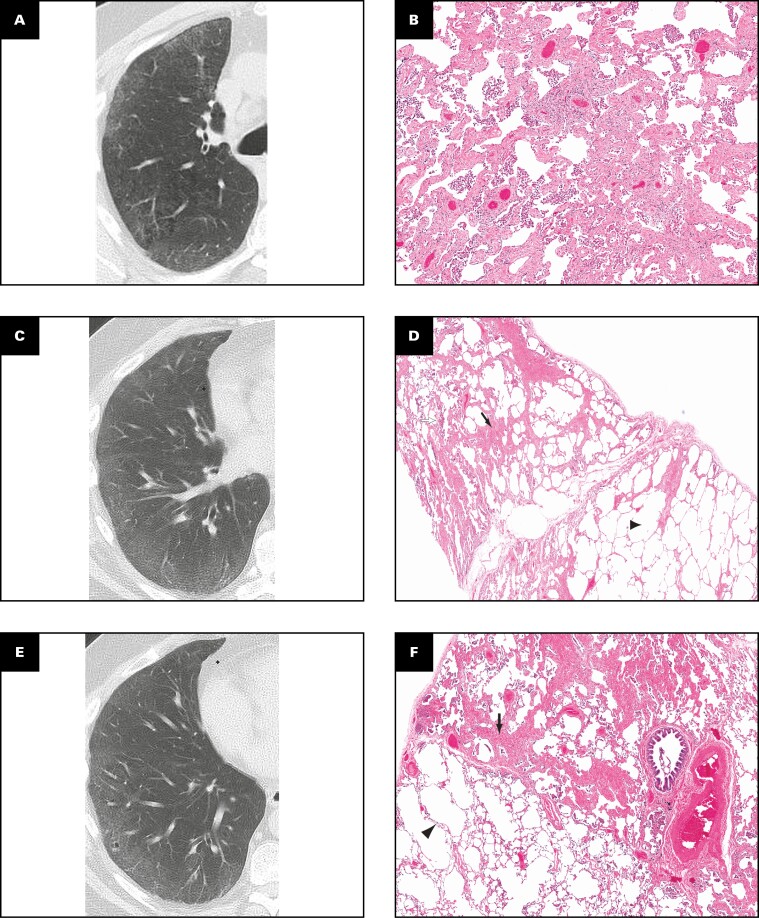
**A-F**, Smoking-related interstitial fibrosis (SRIF): imaging and pathology in upper, middle, and lower lobes. All images are from case 1. **A**, Nonenhanced axial computed tomography (CT) of the right upper lobe demonstrates peripheral ground-glass opacities and trivial centrilobular emphysema. **B**, On pathologic examination, there is diffuse interstitial thickening by fibrosis, mimicking nonspecific interstitial pneumonia (H&E, ×2). **C**, Nonenhanced axial CT of the middle lobe demonstrates subtle peripheral ground-glass opacities. **D**, Pathology of right middle lobe shows patchy SRIF (black arrow). Note alveolar macrophages (white arrow). Lung on the other side of the interlobular septum is normal (arrowhead) (H&E, ×2). **E**, Nonenhanced axial CT of the right lower lobe demonstrates peripheral ground-glass opacities, bronchial wall thickening, and a microcystic space. **F**, SRIF thickens alveolar septa (arrow) in a patchy fashion in the lobule above the interlobular septum. The lobule below the interlobular septum is nonfibrotic (arrowhead) (H&E, ×2).

Our pathologic analysis and scoring were based on surgical lung biopsy specimens. The pathologic findings are shown in correlation with imaging findings in **Figures 4** and **5**. Surgical lung biopsy specimens from all six cases showed SRIF, characterized by thickening of alveolar septa by dense, eosinophilic, “ropey-appearing” hyalinized collagen. Although GGOs were radiologically diffuse, pathologic fibrosis was patchy in most areas, involving mainly subpleural and peribronchiolar lung parenchyma. In some cases, a lobule with SRIF was separated from a lobule of nonfibrotic lung by an interlobular septum FIGURE 5D, 5F. However, occasionally (eg, case 1, upper lobe), fibrosis thickened the alveolar septa in a diffuse and uniform manner, mimicking the histologic appearance of nonspecific interstitial pneumonia (NSIP) FIGURE 5A. Histologically, SRIF involved all lobes but was upper lobe predominant in three cases (cases 1, 2, and 5), middle lobe predominant in one case (case 4), and similar in all three lobes in one case (case 3). In case 6, only the lower lobe was sampled, precluding histologic assessment of distribution. Correlation of histologic SRIF with radiologic GGO using our semiquantitative scoring system is shown in FIGURE 2.

Mild pathologic emphysema was noted in all cases. Notably, subtle pathologic emphysema was present not only in the two cases with radiologic emphysema but also in the four cases in which no emphysema was seen on imaging. Pigmented macrophages (“smokers’ macrophages”) containing fine brown intracytoplasmic pigment with occasional black specks were present within the airspaces in all cases and most numerous in the upper lobes, where they involved more than 75% of the lung parenchyma in five cases and 51% to 75% of the lung parenchyma in one case. Airspace filling by macrophages commonly varied from lobe to lobe and from field to field within the same lobe FIGURE 4D and FIGURE 5D.

Occasional lymphoid aggregates were present within the interstitium in all cases; germinal centers were absent. No fibroblast foci were identified. Scarring (significant architectural distortion by fibrosis) and honeycombing were absent in all but one case (case 6), which contained a single focus of microscopic honeycomb change.

### Longitudinal Evaluation and Care

Follow-up at our institution’s pulmonary clinic ranged from a single office visit to 10 years of longitudinal follow-up TABLE 3. All patients were counseled on smoking cessation. Three patients quit smoking (cases 1, 2, and 4) and remained abstinent except for one patient who used vaping as an alternative. The patient with a single office visit (case 3) was a current smoker at the time of the visit. The other two patients (cases 5 and 6) continued to smoke but cut back consumption by 50% to 75%.

Two patients, one of whom had asthma by medical history, were treated with a combination of inhaled corticosteroids and long-acting β-agonists. One patient was treated with a combination of inhaled long-acting muscarinic agonist with long-acting β-agonist. One patient was treated with oxygen supplementation (case 1) at the time of diagnosis, and two patients (cases 2 and 4) were treated with oxygen supplementation within the follow-up timeline.

Three patients (cases 1, 4, and 6) were treated with a steroid taper over several weeks. Steroid tapers included a maximum of prednisone 20 to 30 mg tapered over the course of 2 to 6 months. Steroids were stopped due to lack of perceived benefit and untoward side effects. No steroid-sparing immunosuppressing agents were used for treatment.

### Serial Spirometry

**FIGURE 6:**
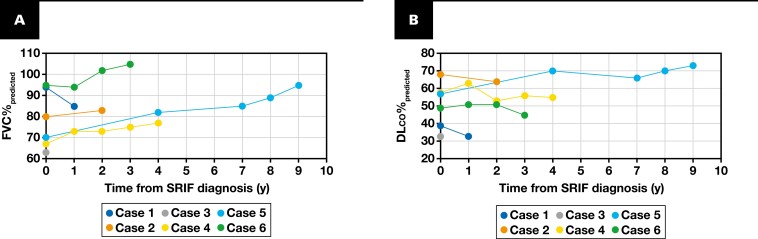
Serial spirometry. **A**, SRIF longitudinal FVC%_predicted_. **B**, SRIF longitudinal DL_CO_%_predicted_. DL_CO_, diffusing capacity for carbon monoxide; FVC, forced vital capacity; SRIF, smoking-related interstitial fibrosis.

Serial spirometry was reviewed if available. The one patient with a single office visit had only one spirometry measurement available for review. The remaining five patients had between 1 and 9 years of spirometry recorded. Longitudinal spirometry data are shown in **FIGURE 6**. None of the patients with serial spirometry available for review had evidence of progression based on spirometry. In patients who quit smoking, the FVC%_predicted_ remained stable within 10% of the initial value and DL_CO_%_predicted_ within 6% of the initial value. In the two patients who continued smoking at a decreased rate (cases 5 and 6), FVC%_predicted_ improved by 25% and 10%, and DL_CO_%_predicted_ improved by 16% or remained stable within 5%, respectively.

### Serial CT Scans

**FIGURE 7:**
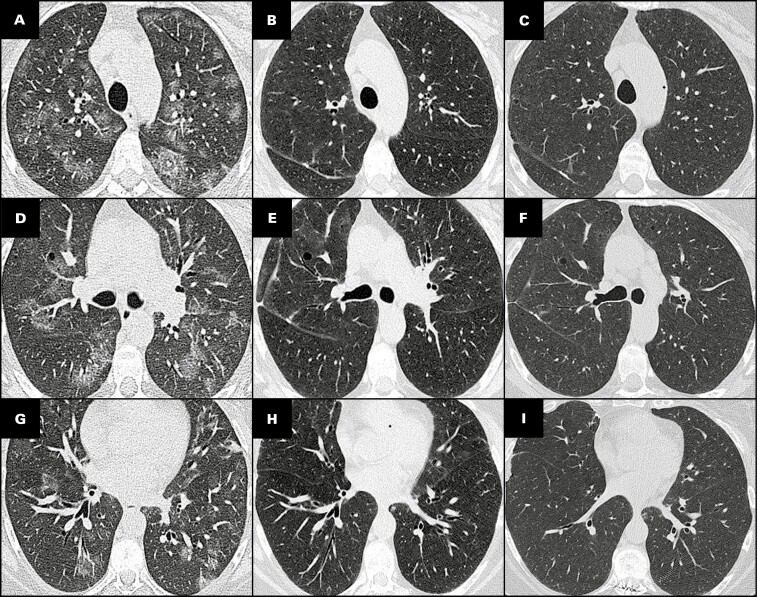
**A-I**, Imaging follow-up in smoking-related interstitial fibrosis. All images are from case 5 (48-year-old woman). **A**, **D**, **G**, Upper, middle and lower lobes from initial computed tomography (CT), showing nodular bronchocentric ground-glass opacities. **B**, **E**, **H**, Upper, middle, and lower lobes from CT at approximately 4 years of follow-up, showing resolution of ground-glass opacities. **C**, **F**, **I**, CT at nearly 8 years (95 months) of follow-up, showing stable findings.

Serial CT scans were reviewed when available. In five patients, GGOs remained stable over follow-up intervals ranging from 3 to 89 months. In the sixth (case 5), in which the patient decreased smoking from 1 to ½ packs per day but did not quit completely, bilateral GGOs resolved on a follow-up CT 4 years after the initial CT and remained stable over a total follow-up period of 95 months **FIGURE 7**.

## DISCUSSION

We describe a retrospective case series of six patients who were referred to our tertiary care DPLD subspecialty clinic with clinical and imaging findings of DPLD, in whom SRIF was diagnosed after surgical lung biopsy and multidisciplinary discussion. All cases came to medical attention due to respiratory symptoms and had reduced pulmonary function and abnormal chest imaging, which prompted invasive biopsy sampling.

A key observation that emerged from the careful rereview of imaging and surgical lung biopsy findings in these cases was that interstitial fibrosis is clearly present in multiple lobes in SRIF (and defines the disease on pathology) but does not correlate with traditional indicators of fibrosis on chest CT such as reticulation, traction bronchiectasis, and honeycomb change. Whether the GGOs in these cases are explained by airspace macrophages, interstitial fibrosis, or both remains unclear. What is clear is that the interstitial fibrosis of SRIF does not cause significant architectural distortion (scarring) and thereby does not manifest radiologically with traditional imaging features of fibrosis. Another surprising observation was that emphysema was present on pathology in some cases but not on imaging likely because mild emphysema falls below the resolution of CT scans.

The imaging findings in this cohort differ from those described in SRIF in lobectomy specimens by Otani et al.^7^ It seems likely that SRIF presenting as DPLD is associated with a different spectrum of features (younger patients, more numerous macrophages, less prominent emphysema, more GGOs) than SRIF that is identified as an incidental finding in lung resections for cancer. In the latter group, features of emphysema appear to be more prominent on both imaging and pathology. Why some heavy smokers develop an emphysema-type phenotype while others develop a ground-glass-type phenotype is unclear and requires further investigation.

This cohort highlights a few recurrent themes that arise in the differential diagnosis of SRIF. The relatively young age of these patients often prompted evaluation for connective tissue diseases. It is important to note that lymphoid aggregates, a pathologic finding often mentioned as a feature of connective tissue disease–related DPLD, were present in all cases, although they were few in number and lacked germinal centers. Given the presence of mediastinal and hilar lymphadenopathy, sarcoidosis was also commonly considered, although atypical features were acknowledged—namely, the presence of GGOs. Finally, SRIF can be misinterpreted as IPF. The young age of these patients with a stable clinical course, imaging features predominantly showing GGOs, an absence of scarring, and lack of fibroblast foci and honeycomb change on pathology should militate against UIP/IPF.

To date, pathologically confirmed SRIF appears to be highly specific for smoking.^1-7^ While it is clear that SRIF is caused by cigarette smoking, the exact pathophysiologic mechanism by which smoking causes this type of interstitial fibrosis remains unknown. It is unclear why some patients with pathologic SRIF have no clinical symptoms while others develop symptoms and imaging features of DPLD. An underlying genetic predisposition or environmental triggering factor may be involved. It is curious that other well-described smoking-related comorbidities such as obstructive airway disease and radiographic emphysema were less pronounced in our SRIF cases. Given the lack of understanding of the pathophysiology, recommendation of meaningful treatment (aside from universally recommended smoking cessation) is challenging.

Reports of prognosis and longitudinal follow-up of SRIF are sparse. Our findings suggest that although symptoms such as dyspnea and cough may persist, SRIF does not progress significantly over up to nearly 10 years of clinical follow-up and may show slight improvements in pulmonary function, as well as long-term stability of radiologic findings with smoking cessation or reduction. We emphasize that clinical or imaging features of progressive pulmonary fibrosis should prompt consideration of alternative diagnoses (such as UIP/IPF), especially in light of increasing data for antifibrotic therapy. Similarly, at a pathologic level, features of UIP such as significant scarring, honeycombing, and/or fibroblast foci should serve as red flags.

The underlying pathologic basis of the improvements seen in some of our cases after smoking cessation remains unclear. It is possible that the improvements may have resulted from a decrease in alveolar macrophages rather than resorption of interstitial collagen deposition. However, in the absence of histologic proof from follow-up biopsies after improvement, this hypothesis remains speculative.

This study has implications on the clinical terminology of smoking-related DPLD. Ideally, any term used for a clinical syndrome should be accurate, encompass the salient pathologic findings, acknowledge the etiology of the process, and be based on the most up-to-date understanding of the disease. There are several possible terms that clinicians use for the clinical disease that occurs in patients with histologic SRIF, including SRIF, RBILD, desquamative interstitial pneumonia (DIP), RBILD/DIP, and the umbrella term *smoking-related interstitial lung disease*. We propose that SRIF can be used as a clinical term, with the caveat that similar to respiratory bronchiolitis (RB), the pathologic features of SRIF can be encountered either as an incidental pathologic finding or as clinical DPLD. Interpreting the pathologic findings in the context of clinical and imaging features will continue to be important in these cases.

Should the cases in this series have been labeled RBILD? While there are similarities in the clinical presentation (young heavy smokers), radiographic findings (GGOs), and outcomes (good) between our cases and RBILD, and all patients had pigmented airspace macrophages, they do not fit with published descriptions of RBILD. In fact, even the term *RB* is problematic. In this article, we used the accurate descriptive term *pigmented airspace macrophages* rather than *RB* since the term *RB* incorrectly suggests that the process is limited to respiratory bronchioles and features an “itis,” both of which are inaccurate for the cases described in this series. In our SRIF cases, pigmented macrophages were predominantly present within alveoli (not respiratory bronchioles) and did not feature chronic inflammation in respiratory bronchioles. Furthermore, in none of our cases was the process bronchiolocentric. Equally importantly, the pathologic findings in our series do not resemble those described in RBILD. In addition to the differing distribution of macrophages, another major histologic difference between SRIF and RBILD was that interstitial fibrosis in our SRIF cases was extensive (in our series, it mimicked NSIP in one case and was misdiagnosed as UIP in another), whereas interstitial fibrosis is absent or at best minimal in RBILD. For example, in the series by Myers et al^11^ of six cases of RBILD, mural fibrosis was seen around membranous bronchioles in only one of six specimens. Similarly, in a 2005 study from the Mayo Clinic, a defining feature of RBILD was the absence of “significant associated interstitial pneumonia.”^12^ In contrast, in our SRIF cases, interstitial fibrosis was extensive and significant. Since RB occurs in virtually all cases of SRIF, it is impossible to prove with certainty whether GGOs on imaging are a consequence of airspace macrophages, interstitial fibrosis, or both. The current dogma—that relies heavily on airspace macrophages to explain GGOs while ignoring interstitial fibrosis—does not explain why most lobectomy cases with significant macrophage accumulation within airspaces (whether limited as in “RB” or extensive as in “DIP”) are completely incidental and do not cause either symptoms or GGOs on imaging.

It can be argued that the clinical, imaging, and pathologic features described here have been previously noted in the subset of DIP that occurs in smokers. Thus, it is likely that these patients would have been labeled DIP by many multidisciplinary teams because many pulmonary pathologists allow some fibrosis in DIP. The question is whether DIP, which is well known to be a suboptimal misnomer,^13^ is an appropriate label for these smoking-related abnormalities or whether a more accurate descriptor should be used. This article describes a subset of smoking-related interstitial lung disease with distinctive and consistent pathologic features and seeks to remove such cases from the broad mixed-bag umbrella of DIP. The problem with using *DIP* as a diagnostic term for smoking-related lung abnormalities is more than simply that the term is a misnomer or that it overlaps substantially with RBILD. The more significant problem with the concept of DIP is that its definition as an entity relies exclusively on macrophages and fails to incorporate other important histologic features (eg, the type and extent of interstitial fibrosis) and significant clinical features (eg, smoking). Defined in this overly simplistic manner, DIP lumps together smoking-related interstitial lung disease (even when it features ropey interstitial fibrosis, is not steroid responsive, and is characterized by long-term clinical stability) with nonsmoking-related entities (a mixed bag of corticosteroid-responsive conditions and other fibrosing interstitial lung diseases) into a single entity.^13^ As currently defined, some cases of DIP are smoking related while others occur in never-smokers. Some are responsive to corticosteroids while others are not. Some remain stable for long periods of time while others progress and lead to death. The resultant situation is analogous to *bronchioloalveolar carcinoma*, a term that was widely used by lung pathologists prior to 2011, even though—like DIP—it lumped together different processes (“mucinous bronchioloalveolar carcinoma” and “nonmucinous bronchioloalveolar carcinoma”) based solely on a lepidic growth pattern. After adenocarcinoma in situ was described by Noguchi and colleagues in 1995,^14^ it gradually became clear that this was a better, more accurate term than bronchioloalveolar carcinoma for a subset of cases because the definition incorporated additional features other than simply a lepidic growth pattern. Similarly, SRIF—which is defined by a histologically distinctive form of interstitial fibrosis that occurs only in smokers—does not replace all cases that would have been previously labeled DIP. However, it is a more accurate term than DIP for cases of interstitial lung disease that occur in smokers and feature ropey interstitial fibrosis on pathology, because the entity defined in this way is more homogeneous in terms of etiology, prognosis, and response to treatment. The term *SRIF* reflects our modern understanding of the clear relationship between smoking and a subset of cases of DPLD, shifts the focus from macrophages to interstitial fibrosis, and extricates these cases from the “idiopathic interstitial pneumonias,” thereby avoiding the oxymoron “smoking-related idiopathic interstitial pneumonia.”

Currently, the umbrella term *smoking-related interstitial lung disease* seems to be acceptable to most clinicians, radiologists, and pathologists for cases that fall under the overlapping spectrum of RBILD, DIP, and SRIF. While smoking-related *interstitial* lung disease may not be a perfect label for rare cases that feature only pigmented (smoking-related) airspace macrophages *without* interstitial fibrosis, it *is* accurate for SRIF, which is defined by the presence of interstitial fibrosis on histology. Whether the distinction between the overlapping categories of RBILD, DIP, and SRIF is clinically significant is worthy of further study.

In this series, the final multidisciplinary diagnosis of SRIF was made only after surgical lung biopsy and multidisciplinary discussion, but it is reasonable to ask whether this diagnosis can be established by transbronchial biopsy. In two of the three cases in this series where a transbronchial lung biopsy was performed, the biopsy specimen showed RB and mild SRIF TABLE 3. The third biopsy specimen was not available for review. Since a confident multidisciplinary diagnosis of SRIF requires that SRIF should be the only explanation for the clinical/imaging findings, this diagnosis is difficult to establish with certainty on transbronchial biopsy alone because of the possibility that a subsequent surgical lung biopsy specimen may reveal histologic findings—such as UIP or pulmonary Langerhans cell histiocytosis—that would override the diagnosis of SRIF and relegate it to an incidental smoking-related finding in the background lung.

Our study has several limitations. This was a small, retrospective study at a single institution with limited follow-up (maximum of 10 years). Because our methodology required histopathology specimens and multidisciplinary discussion at our tertiary center, we inherently selected patients with symptoms and imaging abnormalities that were severe enough to warrant invasive investigation. Furthermore, our cases relied heavily on surgical lung biopsy specimens; interpretation of smoking-related findings in transbronchial lung biopsy specimens is inherently more complicated. Despite the limited generalizability of our study, the increase in widespread availability of CT imaging, the increasing numbers of patients undergoing low-dose CT for lung cancer screening, and increasing familiarity of pathologists with the pathologic features of SRIF could substantially increase the incidence of SRIF in the future.

## CONCLUSIONS

We conclude that SRIF can occasionally present as a clinically meaningful DPLD and is not exclusively an incidental pathologic finding. Patients who have DPLD and show SRIF in surgical lung biopsy specimens are heavy smokers who present most commonly in the fifth decade of life with dyspnea, diffuse bilateral GGOs on chest CT, and reduced DL_CO_ and/or FVC on pulmonary function testing. The clinical course is characterized by relative stability with smoking cessation or reduction, and corticosteroids appear to be ineffective. We prefer the nomenclature of SRIF for this form of smoking-related interstitial lung disease as it clearly states the etiology of the process (smoking) and accurately describes the disease-defining pathologic finding (interstitial fibrosis). While SRIF is most commonly encountered as an incidental pathologic finding in lung resections for cancer, clinicians should remain cognizant of the subset of cases that presents as a clinically meaningful DPLD. More research is needed to further characterize the longer-term clinical implications and optimal management of SRIF.
